# High degree of consensus achieved regarding diagnosis and treatment of acromioclavicular joint instability among ESA-ESSKA members

**DOI:** 10.1007/s00167-020-06286-w

**Published:** 2020-09-26

**Authors:** Claudio Rosso, Frank Martetschläger, Maristella F. Saccomanno, Andreas Voss, Lucca Lacheta, ESA DELPHI Consensus Panel, Ana Catarina Ângelo, Emmanuel Antonogiannakis, Clara Azevedo, Klaus Bak, Semin Becirbegovic, Knut Beitzel, Kerem Bilsel, Roman Brzoska, Angel Calvo, Christophe Charousset, Felix Dyrna, Emmanuel Brilakis, Francesco Franceschi, Jean Marc Glasson, Pascal Gleyze, Nuno Gomes, Roger Hackney, Michael Hantes, Orestis Karargyris, Mustafa Karahan, Ladislav Kovacic, Alexander Kubashev, Lucca Lacheta, Olaf Lorbach, Benjo Maben, Benjamin Marjanovic, Frank Martetschlaeger, Christos Yiannakopolus, Roman C Ostermann, Andreas Panagopoulos, Perikles Papadopoulos, Boris Poberaj, Claudio Rosso, Maristella Francesca Saccomanno, Daniel Smolen, Francesc Soler, Ettore Taverna, Bruno Toussaint, Patrick Vavken, Andreas Voss, Nestor Zurita, Knut Beitzel, Giuseppe Milano

**Affiliations:** 1 ARTHRO Medics, Shoulder and Elbow Center, Thannerstrasse 45, 4054 Basel, Switzerland; 2 University of Basel, Basel, Switzerland; 3 German Center of Shoulder Surgery, ATOS Clinic Munich, Munich, Germany; 4 Department of Sports Orthopedics, Klinikum rechts der Isar, Technical University of Munich, Munich, Germany; 5 Orthopaedics Institute, Fondazione Policilinico Universitario A. Gemelli IRCCS, Rome, Italy; 6 Department of Trauma Surgery, University Medical Center, Regensburg, Germany; 7 Center for Musculoskeletal Surgery, Charitè Universitaetsmedizin Berlin, Berlin, Germany; 8 European Shoulder Associates, European Society of Sport Traumatology, Knee Surgery and Arthroscopy, ESSKA, Luxembourg, Luxembourg; 9 Atos Orthoparc Clinic, Cologne, Germany; 10 Department of Medical and Surgical Specialties, Radiological Sciences, and Public Health, University of Brescia, Brescia, Italy; 11 Department of Bone and Joint Surgery, Spedali Civili, Brescia, Italy

**Keywords:** Acromioclavicular joint, Ac joint instability, Instability, Ac joint, Treatment, Diagnosis, Consensus, Delphi, European shoulder associates

## Abstract

**Purpose:**

To develop a consensus on diagnosis and treatment of acromioclavicular joint instability.

**Methods:**

A consensus process following the modified Delphi technique was conducted. Panel members were selected among the European Shoulder Associates of ESSKA. Five rounds were performed between October 2018 and November 2019. The first round consisted of gathering questions which were then divided into blocks referring to imaging, classifications, surgical approach for acute and chronic cases, conservative treatment. Subsequent rounds consisted of condensation by means of an online questionnaire. Consensus was achieved when ≥ 66.7% of the participants agreed on one answer. Descriptive statistic was used to summarize the data.

**Results:**

A consensus was reached on the following topics. Imaging: a true anteroposterior or a bilateral Zanca view are sufficient for diagnosis. 93% of the panel agreed on clinical override testing during body cross test to identify horizontal instability. The Rockwood classification, as modified by the ISAKOS statement, was deemed valid. The separation line between acute and chronic cases was set at 3 weeks. The panel agreed on arthroscopically assisted anatomic reconstruction using a suspensory device (86.2%), with no need of a biological augmentation (82.8%) in acute injuries, whereas biological reconstruction of coracoclavicular and acromioclavicular ligaments with tendon graft was suggested in chronic cases. Conservative approach and postoperative care were found similar

**Conclusion:**

A consensus was found on the main topics of controversy in the management of acromioclavicular joint dislocation. Each step of the diagnostic treatment algorithm was fully investigated and clarified.

**Level of evidence:**

Level V.

## Introduction

Injuries of the acromioclavicular (AC) joint are quite common, accounting for 3–12% of all shoulder injuries [9]. The incidence even rises up to 40–50% when it comes to contact sports [14], with the highest prevalence in men in their second or third decade of life [7]. It seems evident that diagnosis and management of acute and chronic AC joint dislocations need to be well stated. However, although a plethora literature is available, a clear consensus has still not been achieved.

Traditionally, AC joint dislocations have been diagnosed on radiographs, through bilateral standard anteroposterior (AP) and Zanca views, and then classified according to the Rockwood classification. Conservative management is usually preferred in low-grade injuries (Rockwood type I and II), whereas symptomatic high-grade injuries (types IV–VI) are routinely managed surgically. Management of acute type III injuries is still an ongoing subject of controversy [8, 11, 19]. Decision making is often based on patient’s work and sporting activity as well as surgeon’s personal opinion and experience. However, concerns have been raised on each step of the decision-making process. Even when it comes to conservative management, best type and length of immobilization have not been defined yet [24]. Regarding surgical therapy, the wide range of available new surgical procedures clearly reflects the lacking of a golden standard; each technique is associated with limitations and, finally, none of them have been demonstrated to be superior to the others with respect to clinical outcomes [5, 24].

Therefore, the European Shoulder Associates (ESA), section of the European Society of Sports Traumatology, Knee Surgery and Arthroscopy (ESSKA), aimed to develop a consensus on the evaluation and management of AC joint dislocation to provide a unified expert opinion on this topic. It was hypothesized that there would be a high degree of consensus in the diagnosis and the treatment of AC joint dislocations despite the plethora of literature on diagnostic tools and treatment options.

## Materials and methods

A consensus process with an international panel of experienced clinicians using the modified Delphi technique was implemented [1, 13].

The Delphi procedure is a systematic instrument, which aims to measure and develop consensus when empirical evidence is lacking. The ESSKA-ESA followed the steps of this procedure to guarantee the quality of its work.

The process consisted of two consecutive phases: systematic literature reviews and consensus development.

### Systematic review

The systematic literature reviews of imaging and treatment were published in 2018 [18]. These publications were made available for the questions of the Delphi consensus.

The results of the literature search were then allocated according to the three following items: imaging; classification; and treatment. All search results not allocated to the above were not considered for further evaluation.

### Consensus development

According to Hsu et al. [13] and Audige et al. [1], the Delphi consensus was developed. Criteria for not further asking a question in the next round were: (a) ≥ 66.7% of the participants agreed on one answer; (b) The percentage of the answer was steady between two rounds; c) If no consensus was found in round 5, this question was marked as having “no consensus” for any of the answers.

In total, 5 rounds were performed within 18 months of the Delphi process (systematic reviews in May 2018, round 1 in October 2018, round 5 in November 2019). Round 1 consisted of a panel meeting at the ESA closed meeting in Athens, October 2018. Rounds 2 to 4 were based on online questionnaires. Round 5 was a panel meeting at ESSKA Specialty Days, Madrid, November 2019.

If an answer had not reached consensus within one round, the panel was informed about the percentage on respondent voting for the according answer. Suggestions for new answers were implemented in the next round in rounds 2 to 4. Each round was prepared by the main and senior authors, who remained blinded to respondent identities when reviewing responses.

### Nomination and selection of panel members

Panel members were selected among the members of ESA for rounds 2 to 4. For round 1 and 5 the panel was made up by the auditorium willing to participate. For round 5, two participants were chosen to be vote counters. Respondents to either of rounds 2 to 4 were considered panel members and were invited to participate in the final, fifth Delphi round.

### Round 1: Development of initial questions and answers (Q&A)

After systematically reviewing the current literature and evidence, important questions and possible answers regarding the diagnosis and treatment of ACJ separation were gathered in and open panel meeting in round one. **CR** and **KB** lead the panel meeting and collected the Q&A. The panel was confronted with the current evidence. If an answer was supported by current literature, it was noted for round two.

### Round 2: Gathering additional Q&A

The Q&A of round one was entered into an electronic data-capture system (Google Forms, Google Inc., Alphabet Inc., Mountain View, CA, USA). The panel was able to review the current literature on each question and have an informed answer on all the questions. Answers for open questions were noted to round three.

### Rounds 3 and 4: Condensing

Answers from round 2 were assessed by the core panel (CR, KB, FM, GM) for the above-mentioned criteria (agreement ≥ 66.7% (consensus level) steady percentage between two rounds). If an answer reached the consensus level, it was not asked again in the following round.

### Round 5

The answers that either did not reach a consensus level or unclear questions were discussed in an open panel meeting. If a consensus was found, it was noted accordingly.

### Statistical analysis

Survey data were transferred to SPSS Statistics 25 software (IBM Corp., Armonk, NY, USA) for standard descriptive analyses. Consensus was achieved for a categorical response when it involved at least two-thirds of respondents. Final adjudication after the fifth survey was made by the authors for a few questions that did not lead to a clear consensus.

## Results

From this first round, members were asked to participate in the consensus process. In the second round, 28 out of 49 (57%) responded, in the third round 29 (59%), and 30 (61%) in the fourth round. At the final round, which was again not online, 40 panel members were available for voting. Before opening the consensus questions, the panel was asked about their frequency of annual AC-joint surgeries. About 54% treated between 10–50 AC joints, whereas the other 46% treated less than 10 AC joints per year (Table 1).

**Table 1:** Average annual AC joint surgeries

Number	Percentage
< 10	46.4%
10–30	39.3%
31–50	14.3%

Questions were divided into 5 blocks referring to the radiographic modalities to diagnose AC joint pathologies, the classification systems to grade differences, the surgical approach for acute and chronic cases as well as the postoperative treatment.

### Radiographic evaluation

**Fig. 1:**
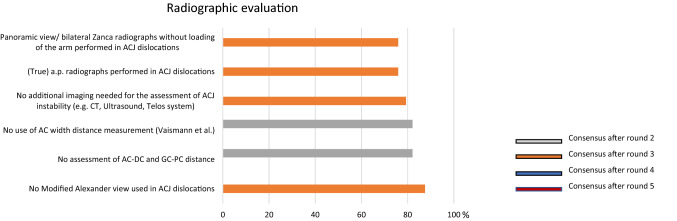
Graphical illustration of radiographic evaluation. Consensus was found after round 2 and 3, respectively. Panoramic views with true a.p. radiographs without additional imaging (MRI, CT, etc.) were found to be sufficient

After the final round, the panel reached a consensus regarding the radiological approach to diagnose and classify AC joint dislocations. The consented radiographs are a true a.p. radiograph, as well as a panoramic view (bilateral Zanca radiographs) without loading of the arm. To address the horizontal instability through radiographs no consensus was reached. However, clinical override testing during body cross test was proposed by 93% of the panel members to identify horizontal instability. In addition, a consensus was reached after the third round (79.3%), that no additional imaging is needed for the assessment of AC joint instability (e.g. computed tomography, magnetic resonance or ultrasound, Fig. 1).

### Classification

**Fig. 2:**
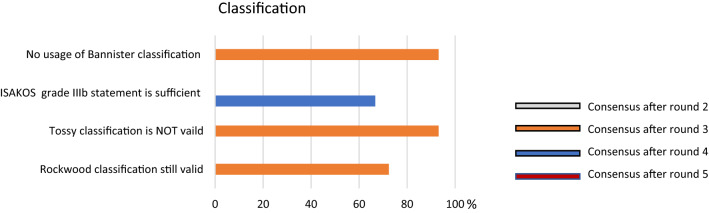
Graphical illustration of the classification system. Consensus was found after round 3 and 4, respectively. The Rockwood classification was recommended by the panel

After round three there was a clear consensus regarding different classifications. The Tossy classification [26] and the Bannister classification [2] are not recommended to classify the type of AC dislocation (93.1% respectively 93.10% voted against using this classification). So far, the Rockwood classification is still the most valid classification. The ISAKOS statement (concerning grade III) was consented to be sufficient for a comprehensive classification (Fig. 2).

### Acute injury

**Fig. 3:**
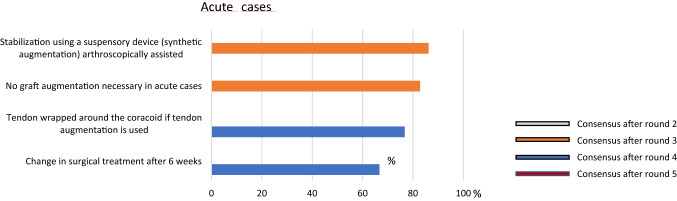
Graphical illustration of acute cases. Consensus was found after round 3 and 5, respectively. It was consented to use a stabilization with a suspensory device in acute cases

After round four an acute case was defined as an AC joint dislocation presenting within the first 3 weeks after trauma. Regarding the surgical treatment, an arthroscopically assisted anatomic reconstruction using a suspensory device (synthetic augmentation) is recommended (86.2%), with no need of an additionally biological augmentation (82.8%) (Fig. 3).

### Chronic injury

**Fig. 4:**
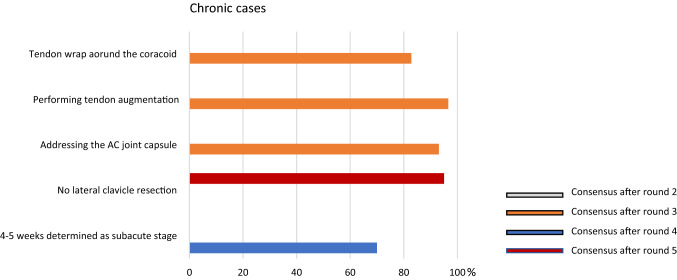
Graphical illustration of chronic cases. Consensus was found after round 3, 4 and 5, respectively. It was consented to use a tendon augmentation wrapped around the coracoid and addressing the AC joint capsule

As following the definition of acute cases, the panel defined a chronic case if the initial trauma occurred more than 3 weeks ago. There was an early (after round 3) consensus regarding the usage of biological augmentation in chronic cases, with the need to address the AC capsule. Therefore, a tendon augmentation is recommended, wrapping the tendon around the coracoid. Additionally, there is no recommendation for a distal clavicle resection in chronic cases (95%) and the panel denied using this surgical approach (Fig. 4).

### Treatment

**Fig. 5:**
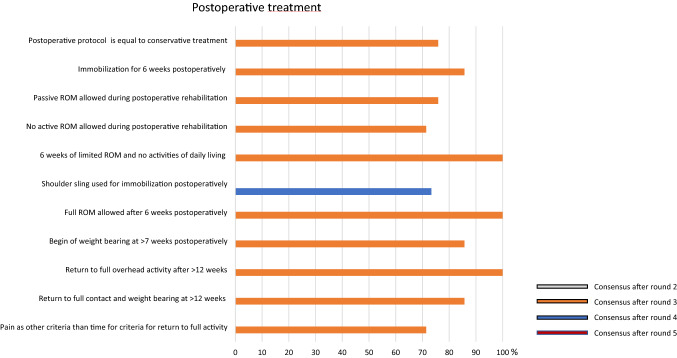
Graphical illustration of postoperative treatment. Consensus was found after round 3 and 4, respectively

Postoperative treatment modalities differed depending on acute or chronic cases. The results showed no different treatment strategy of conservative or postoperative treatment, in regard to “back-to-sports”, weight restrictions or active and passive mobilization. A shoulder sling is recommended for immobilization for 3 weeks after surgery. A high consensus was reached (100%) with a limitation of range of motion with no activities of daily living for the first 6 weeks and a free range of motion 6 weeks after surgery (100%) (Fig. 5).

The mode to change from conservative to surgical treatment is defined by the patients´ persistence of pain (93.3%). Additionally, weight restrictions are cleared after 3–4 months (90%).

## Discussion

The most important finding of the present study was that finally a consensus could be found on several topics. True AP view or a bilateral Zanca view were deemed sufficient for diagnosis, a separation line between acute and chronic was set at 3 weeks from trauma, arthroscopically assisted anatomic reconstruction using synthetic augmentation has been suggested in acute injuries, whereas the use of biological reconstruction with tendon graft was reserved to chronic cases. Clarification on postoperative protocol and conservative management have also been made.

Since AC joint dislocations are plagued by limited evidence-based literature, the present consensus really shed light on some controversial issues.

An emerging concept in the quest for a better understanding of AC joint pathology and improved clinical outcomes is the complementary role of either coracoclavicular (CC) and AC ligaments. Decades ago, biomechanical studies have clearly stated that CC ligaments are the main responsible for vertical stability, as well as AC ligaments and capsule are the primary stabilizers in the horizontal plane [4, 11]. Since clinical data has shown a vast number of persistent horizontal instability following modern arthroscopic AC joint reconstruction techniques [23, 24], not only the surgical management but also the clinical assessment have been reconsidered.

It has been claimed that parameters assessable on AP and Zanca view do not allow for quantification of horizontal instability, therefore, the use of new radiographic parameters in a single lateral Alexander view has been recently recommended [15, 29]. Anyhow, the ESA panel agreed that a true AP view or a bilateral Zanca view without loading the arm are still adequate for a correct diagnosis, with no need of modified Alexander view to seek for horizontal instability. On the contrary, the clinical evaluation was deemed sufficient to evaluate instability in the horizontal plane.

Similarly, recent papers called into question the reliability of the Rockwood classification [16, 21] and further stated that, except for type IV, it does not assess horizontal instability [29]. Once again, the consensus clarified that the Rockwood classification, recently modified by the ISAKOS statement [3], remains the most appropriate and comprehensive classification to guide the treatment choice so far.

Moving forward, once the diagnosis has been made and the dislocation has been correctly classified, current literature turned out to be unable to provide a clear demarcation line between acute and chronic dislocations. While some authors considered acute dislocations those treated within 3 days after trauma [12, 27], some others still considered acute injuries those treated up to 4–6 weeks after trauma [6, 8, 17, 28]. The ESA panel unequivocally set the separation line at 3 weeks, but also defined a grey zone between acute and chronic ranging from 3 to 6 weeks. According to the ESA panel this should be considered an important turning point when it comes to surgical management. Taking into account the limited healing capacity of both CC and AC ligaments, definition of chronic setting clearly affects the surgical strategy. As a matter of fact, a large consensus stated that arthroscopically-assisted reconstruction using a suspensory device (synthetic) with no need for an additional biologic augmentation should be the treatment of choice in acute cases, whereas biologic reconstruction to re-create not only CC ligaments but also AC ligaments was deemed necessary in chronic cases. In other words, the less healing response is expected, the more surgical stability, increased by biological augmentation, is recommended. Moreover, biomechanical studies demonstrated that combined AC and CC ligaments reconstruction provides better results than isolated CC reconstruction [10, 22].

Conservative treatment is once again an unclarified issue. It generally involves immobilization of the arm. Several types of arm immobilizers have been proposed [25] ranging from a broadarm sling up to Kenny–Howard splint, taping and casts. Immobilization can last from 3 days up to 3 weeks based on Rockwood type, subsiding pain and/or different protocols available [20, 24]. Rehabilitation starts gradually after sling removal. Unfortunately, no previous studies aimed to clarify whether one immobilizer is better than the other neither if a longer period of immobilization has a biologic rationale, therefore, the final decision is always up to the surgeon’s experience. The ESA panel aimed to summarize the current literature, thus providing a sort of reasonable guideline to follow and a consensus has been reached on this topic. Conservative management of low-grade AC joint dislocations was unified with postoperative management of high-grade AC joint dislocations. Three weeks of immobilization seemed a reasonable time to provide an initial biological ingrowth, thus avoiding risks related to a longer immobilization period (e.g. shoulder stiffness). However, 6 weeks are warranted before regaining full range of motion and activities of daily living. Sports activities are not allowed before 4 months. Anyhow, according to the ESA panel, pain still remains the main criteria for return to full activities as well as to switch a conservative management into a surgical one.

Nevertheless, some issues still remain controversial and represent the limitations of the present study. One for all, outcome measures to evaluate the management of ACJ injuries are not consistently reported in the literature, therefore, they could not even be included in the consensus process. Further, the lack of uniformity in reported outcomes and the abundance of conservative treatment protocols as well as surgical techniques reported in the literature make any kind of comparison difficult or somehow inconclusive.

Due to the lack of prospective randomized trials, this consensus statement is meant to be a guideline to get insight into the complex topic of diagnosis and treatment of AC joint dislocations for the general orthopaedic surgeon and even for shoulder specialists, respectively.

## Conclusions

A consensus was reached on main topics of controversy. True AP view or a panoramic view (bilateral Zanca radiographs) without loading of the arm was deemed sufficient for diagnosis. Horizontal instability can be identified through clinical override testing during body cross test. The Rockwood classification, as modified by the ISAKOS statement, is still considered the most valid so far. The separation line between acute and chronic cases was consensually set at 3 weeks. Arthroscopically assisted anatomic reconstruction using a suspensory device (synthetic augmentation) with no need of an additionally biological augmentation could be recommended in acute injuries, whereas the use of biological reconstruction with tendon graft should be preferred in chronic cases, with the need to address horizontal instability by reconstructing also the AC ligaments. Finally, the consensus showed no different treatment strategies between conservative and postoperative care of high-grade ACJ dislocation, recommending immobilization for 3 weeks with a full range of motion activity allowed after 6 weeks.

The ESSKA-ESA section tried to fully investigate and clarify each step of the diagnostic treatment algorithm, aiming to give surgeons insight into the current concepts suggested despite the large amount of literature.
